# A novel recombinant oligogalacturonide lyase from *Klebsiella variicola* promotes pectin degradation and enhances aroma formation in tobacco leaves

**DOI:** 10.3389/fbioe.2026.1824056

**Published:** 2026-06-16

**Authors:** Shen Huang, Caiping Chang, Aamir Rasool, Robina Manzoor, Chao Liu, Ruoxin Wu, Jialiang Wang, Duobin Mao

**Affiliations:** 1 College of Tobacco Science and Engineering, Zhengzhou University of Light Industry, Zhengzhou, China; 2 Institute of Biochemistry, University of Balochistan, Quetta, Pakistan; 3 Department of Biotechnology and Bioinformatics, Lasbela University of Agriculture, Water and Marine Science, Uthal, Pakistan; 4 Tianjin Work Station, Technical Center, Shanghai Tobacco Group Co., Ltd., Tianjin, China

**Keywords:** enzymatic properties, fermentation, heterologous expression, oligogalacturonide lyase, pectin, pectinases, tobacco extract

## Abstract

Microbial enzymes can be exploited to improve the sensory quality and reduce the harmful effects of tobacco-originated compounds in tobacco-derived products. In this study, a novel oligogalacturonide lyase (OGL) gene from *Klebsiella variicola* GB3 was cloned and heterologously expressed in *Escherichia coli* BL21(DE3). The recombinant enzyme was purified to a protein concentration of 18.63 mg/mL. OGL exhibited the highest activity toward tetragalacturonic acid (GalA_4_), with kinetic parameters of *K*_*m*_ = 3.89 ± 0.18 mM and *V*_max_ = 36.41 ± 1.20 μmol/min/mg, and optimal catalytic activity at 45 °C and pH 6.0. The enzyme remained stable over a moderate temperature range (30–40 °C) and a broad pH range (6.0–8.0), while Ca^2+^ significantly enhanced its catalytic activity. The treatment with 1% OGL degraded approximately 32.18% of tobacco pectin, accompanied by significant increases in galacturonic acid (39.86%) and oligogalacturonides (403.85%). Sensory evaluation indicated improved aroma quality, increased smoothness, and reduced irritation in OGL-treated tobacco strands. GC-MS analysis confirmed that OGL treatment significantly enhanced the production of key aroma compounds, including norisoprenoids, furans, and terpenoids, while reducing undesirable off-flavor compounds. Collectively, these results indicate that OGL efficiently degrades tobacco pectin, promotes the release of aroma precursors, and the formation of key flavor compounds. This study highlights the potential of OGL as a promising biocatalyst for improving aroma quality and enabling the value-added bioconversion of tobacco resources.

## 1 Introduction

Tobacco (*Nicotiana tabacum*) is a valuable crop (Xi et al., 2025), which contains more than 4,000 chemical constituents (Farshid et al., 2021; Tsubasa et al., 2023). The quality and sensory characteristics of tobacco products largely depend on the chemical composition of the leaf matrix and the biochemical transformations that occur during curing and processing.

Pectin is one of the most complex groups of polysaccharides present in the plant cell wall and represents a major structural component of tobacco tissues. The degradation of pectic substances plays an important role in modifying cell wall structure and producing tobacco flavor and aroma compounds. Pectin degradation is mediated by pectinases, including protopectinases, polygalacturonases, pectin lyases, and pectin esterases (John et al., 2020; Zheng et al., 2021; Lambré et al., 2022). Among these enzymes, oligogalacturonide lyase (OGL) plays a key role in the degradation of oligogalacturonides (Hobbs and Boraston, 2024). OGL catalyzes the cleavage of α-1,4-glycosidic bonds via a β-elimination mechanism in oligogalacturonides and produces the unsaturated oligogalacturonide products (Zhou et al., 2017). Tobacco leaves contain a complex mixture of chemical constituents that can play a role in the irritation and harshness of tobacco products. Enzymes involved in pectin degradation may enhance the liberation of carotenoid- and terpenoid-derived intermediates, which are subsequently converted into volatile aroma compounds.

Several studies have reported that enzymatic treatment of tobacco can increase the formation of important aroma compounds, including solanone (sweet tobacco-like aroma), dihydroactinidiolide (tea-like and woody fragrance), β-damascenone and β-ionone derivatives (floral and fruity notes), furfural (sweet caramel-like aroma), and other norisoprenoid compounds (Yang et al., 2022; Weng et al., 2024; Sun et al., 2024). Consequently, the application of specific enzymes such as OGL may not only promote pectin degradation but also stimulate the formation of desirable volatile metabolites while reducing the accumulation of certain undesirable compounds (Haile and Ayele, 2022).

Advances in genetic engineering and recombinant DNA technology have enabled the efficient production of industrial enzymes using microbial expression systems (Tian et al., 2022; Chen et al., 2024). *Escherichia coli* has become one of the most widely used hosts for heterologous protein expression because of its rapid growth rate, well-characterized genetics, and suitability for large-scale fermentation (Jia and Jeon, 2016; Yan et al., 2023). Although several pectin lyases from different microorganisms have been cloned and characterized, significant diversity exists in their catalytic properties, substrate specificity, and industrial applicability (Shevchik et al., 1999; Abbott et al., 2010). Despite the recognized industrial potential of OGL enzymes, to date, no studies have reported the cloning, heterologous expression, or functional characterization of OGL from *K. variicola*. Understanding the catalytic properties and potential applications of this enzyme could provide new opportunities for improving tobacco processing and aroma production.

In this study, we aimed to: (1) clone and heterologously express the ogl gene from *K. variicola* GB3 in *E. coli*; (2) perform comprehensive biochemical characterization of the recombinant OGL; (3) evaluate the enzyme’s ability to degrade tobacco pectin; and (4) assess the impact of OGL treatment on aroma compound formation and sensory quality in tobacco strips and extracts.

## 2 Materials and methods

### 2.1 Strains, plasmids, and reagents

The target gene *ogl* (ENA: OZ351899) was sourced from *K. variicola* GB3, a pectinase-producing strain. The gene encoding the enzyme OGL was identified by genomic sequencing and was significantly upregulated in transcriptomic analysis when GB3 utilized tobacco pectin. Its high expression level under these conditions was verified by real-time quantitative fluorescence PCR (RT-qPCR). Recombinant *E. coli* BL21(DE3) competent cells and the prokaryotic expression vector pET-28a were provided by Beijing Bomeite Biotechnology Development Co., Ltd. Primer synthesis and DNA sequencing services were also carried out by the same company. A protein marker ranging from 14.4 kDa to 116.0 kDa was purchased from Thermo Fisher Scientific. IPTG, kanamycin sulfate, and tris (hydroxymethyl) aminomethane (Tris) were obtained from Sangon Biotech (Shanghai) Co., Ltd. A 5 mL Ni-NTA His-tag affinity purification column was purchased from Qiagen (Germany). Trigalacturonic acid (GalA_3_) and tetragalacturonic acid (GalA_4_) were obtained from Shanghai Yuanye Bio-Technology Co., Ltd. The tobacco pectin used in this study was extracted from a blend of ten distinct varieties of flue-cured tobacco leaves using an ultrasonic-assisted method, with a detailed protocol provided in the Supplementary Material S1. The tobacco extract was prepared through pretreatment, solid-liquid separation, and concentration, as detailed in Supplementary Material S2. Luria-Bertani (LB) medium was used for bacterial cell culture.

### 2.2 Construction of the expression vector for *OGL*

The *ogl* gene encodes a protein of 391 amino acids (∼1,173 bp coding sequence). It was amplified from the genome of *K. variicola* GB3 using the following primers: F: 5′-[NcoI: CCA​TGG]-CAT​CGC​AAC​TAC​TTC​TAT-3′; R: 5′-[XhoI: CTC​GAG]-CGA​TAT​TCA​GCA​GAT​AGT​A-3, containing NcoI/XhoI restriction sites (Supplementary Material S3). The *ogl* was inserted into the kanamycin-resistant pET-28a vector using T4 DNA ligase (Zhao et al., 2018; Tian et al., 2022). The recombinant plasmid was then transformed into *E. coli* BL21(DE3) competent cells and plated on LB agar containing 50 μg/mL kanamycin, followed by overnight incubation at 37 °C. Positive clones were confirmed by colony PCR and DNA sequencing and stored at −80 °C for future use.

### 2.3 Heterologous expression of OGL in *Escherichia coli*

The recombinant *E. coli* BL21(DE3) cells carrying the pET-28a-*ogl* construct were inoculated into LB liquid medium with kanamycin at a final concentration of 50 μg/mL. The cultures were grown at 37 °C with shaking overnight. Next, the cultures were transferred to 1 L of LB medium and incubated at 37 °C until the optical density at 600 nm (OD_600_) reached approximately 0.8. IPTG was added at a final concentration of 1 mM to the cell cultures to induce the expression of *ogl* gene. The cell culture was incubated at 16 °C for 16 h. Afterwards, the cells were harvested by centrifugation at 4 °C and 4,000 × *g* for 30 min. The cell pellet was washed twice in sterile water and then stored at −20 °C for later use. These conditions were selected following systematic optimization of IPTG concentration (0.1–1 mM) and induction temperature (16 °C–37 °C). The combination of 1 mM IPTG and 16 °C for 16 h consistently yielded the highest amount of soluble, active OGL, with minimal inclusion body formation.

### 2.4 Purification of recombinant OGL and SDS-PAGE analysis

The collected bacterial cells were resuspended in lysis buffer (20 mM Tris-HCl, 150 mM NaCl, pH 7.5) at a ratio of 1 g of cells to 15 mL of buffer. Cell disruption was carried out using an ultrasonic homogenizer under ice-cold conditions with the following settings: 60% power, alternating 5 s on and 5 s off, for 20 min of sonication. After sonication, the lysate was centrifuged at 4 °C and 10,000 × *g* for 15 min. The resulting supernatant, containing the crude enzyme extract, was collected for further purification.

The crude enzyme was purified using a Ni-NTA affinity chromatography column with an ÄKTA purifier system. The concentrations of the buffers and reagents used for the ÄKTA purification system were as follows: Binding buffer: 20 mM Tris-HCl (pH 7.5), 500 mM NaCl, 20 mM imidazole; Washing buffer: 20 mM Tris-HCl (pH 7.5), 500 mM NaCl, 20 mM imidazole; Elution buffer: 20 mM Tris-HCl (pH 7.5), 500 mM NaCl, 1 M imidazole.’ Protein elution was performed using a linear gradient of elution buffer from 0% to 20% Buffer B, corresponding to an imidazole gradient of 0–200 mM. The purification system was equilibrated with binding buffer at a pressure of 0.3 MPa and a flow rate of 3.0 mL min^−1^ until baseline stability was achieved. Protein elution was carried out using a gradient of elution buffer, and the target fraction containing OGL was collected. The eluted protein fraction was desalted using a HiTrap™ Desalting column to remove imidazole and other salts. The resulting purified protein solution was stored at 4 °C, ready for subsequent activity assays and application studies.

Protein purity was evaluated by SDS-polyacrylamide gel electrophoresis (SDS-PAGE) using a 10% resolving gel and a 5% stacking gel (Matsumoto et al., 2019; Kielkopf et al., 2021). Protein concentration was measured using the bicinchoninic acid (BCA) assay (Cortés-Ríos et al., 2020; Rogatsky, 2021), employing bovine serum albumin (BSA) as the standard reference.

### 2.5 Characterization of the enzymatic properties of recombinant OGL

The enzymatic activity of recombinant OGL was determined by spectrophotometry as described by Zhao et al. (2018), Liu et al. (2024). The enzyme assay was performed in a total reaction volume of 4 mL in a 1-cm cuvette, consisting of 20 µL of OGL working solution (5 mg/mL, diluted from 18.63 mg/mL stock; final enzyme concentration in the reaction: 0.025 mg/mL), 1,980 µL of 10 mM Tris-HCl buffer (pH 7.5), and 2,000 µL of substrate solution (1 mg/mL; final substrate concentration in the reaction: 0.5 mg/mL). The reaction was terminated by heating at 100 °C for 10 min and subsequently cooled to room temperature under running water. Substrates that produce unsaturated products such as tobacco pectin, pectin, trigalacturonic acid (GalA_3_), tetragalacturonic acid (GalA_4_), polygalacturonic acid (PGA) were measured in absorbance at 235 nm, and substrates that release 2-nitrophenol (PNP) such as 4-nitrophenyl-β-D-galactoside (PNPG^1^), 4-Nitrophenylα-D-galactopyranoside (PNPG^2^), 2-Nitrophenyl β-D-glucuronide (PNPG^3^) and 4-Nitrophenyl-β-D-glucopyranoside (PNPG^4^) were measured in absorbance at 405 nm. An equal volume of heat-inactivated OGL solution served as the blank. Standard curves were prepared using authentic 4,5-unsaturated digalacturonate or PNP. Definition of Enzyme Activity: One unit (U) of enzyme activity is defined as the amount of enzyme required to release 1 µmol of product per minute under the assay conditions. Specific enzyme activity is expressed as U/mg.

Enzyme activity calculation formula: Specific activity (U/mg) = (ΔA × V × 10^3^)/(ε × t × m). Where: ΔA: Change in absorbance (Sample Absorbance − Blank absorbance); V: Total reaction volume (L); ε: Molar extinction coefficient of the product (L/(mol·cm)), ε_235_ nm = 4600 L/(mol·cm), ε_405_ nm = 18,000 L/(mol·cm); t: Reaction time (min); m: Mass of enzyme protein in the reaction system (mg).

#### 2.5.1 Substrate specificity analysis

To determine the substrate specificity of the recombinant OGL, various substrates were used at a concentration of 1 mg/mL. Depending on the substrate type, reactions were conducted in a pH 7.5, 10 mM Tris-HCl buffer at 40 °C for 30 min, and the absorbance of the reaction mixture was measured at either 235 nm for unsaturated products or 405 nm for PNP release. Since the activities obtained by the 235 nm and 405 nm methods are not directly comparable, the substrate exhibiting the highest activity in each assay was defined as 100%, and the relative activities of other substrates were calculated accordingly.

#### 2.5.2 Determination of temperature and pH

The optimal temperature for recombinant OGL was identified by measuring enzyme activity across a temperature range of 30–70 °C at 5 °C intervals using the preferred substrate, with activity normalized to the maximum value (100%). Afterwards, pH optimization was conducted at the determined optimal temperature using 10 mM Tris-HCl buffers with pH values ranging from 3.0 to 11.0 at 1-unit intervals, following the same assay protocols.

#### 2.5.3 Stability assessment

Thermal stability was assessed by incubating the enzyme in buffer at temperatures from 20 °C to 80 °C for 0.5–3 h, then measuring the residual activity at each time point. Similarly, pH stability was tested by incubating the enzyme in buffers with pH values ranging from 5.0 to 9.0 for 0.5–2 h, followed by measurement of residual activity. The thermal stability of the OGL enzyme was further quantified by calculating its half-life (t_1/2_) through nonlinear curve fitting of the stability data.

#### 2.5.4 Effect of metal ions

To examine the impact of metal ions on recombinant OGL activity, the enzyme was incubated in buffer solutions containing different metal ions (mM concentrations) at the optimal pH for 30 min at 45 °C. Enzymatic activity was then assessed under the optimal temperature conditions. A reaction mixture without metal ions served as the control.

#### 2.5.5 Kinetic parameter determination

Enzymatic activity was measured using GalA_4_ at concentrations ranging from 0 to 5 mg/mL (corresponding molar concentrations used for Km calculation) under optimal pH and temperature conditions. The maximum reaction velocity (*Vmax*) and Michaelis constant (*Km*) were determined by non-linear regression fitting of the Michaelis–Menten equation using Origin software. The equation is expressed as: *V* = (*V*_max_ × [*S*])/(*K*_*m*_ + [*S*]). Where *V* is the initial reaction rate and [*S*] is the substrate concentration.

### 2.6 Application study of recombinant OGL

#### 2.6.1 Sensory evaluation of tobacco leaves treated with recombinant OGL

500 g flue-cured tobacco leaves (Zhongyan 100 variety) with midribs removed were loosened, conditioned to appropriate moisture, and then cut into fine strips. 50 g tobacco strips were accurately weighed and placed into a sterile incubation bag. An enzyme preparation was formulated by dissolving the desalted OGL to a concentration of 5 mg/mL. The enzyme was applied at a dosage of 1% (w/w; enzyme protein mass to tobacco mass), using a total spray volume of 10% (v/w; solution volume to tobacco mass). This corresponded to spraying 5 mL of enzyme solution (containing 25 mg OGL) onto 50 g tobacco strips using a sterile spray bottle, while the control group was sprayed with an equal amount of distilled water. The treated leaf strips were sealed and incubated at 45 °C for 6 h. The moisture was reduced to 12% by drying the samples in an oven set at 85 °C. Afterward, treated leaves were rolled into cigarettes and conditioned for 48 h under controlled relative humidity (60% ± 2% RH) and temperature (22 °C ± 1 °C).

The sensory evaluation was conducted using a double-blind design. A panel of seven experts with over 5 years of experience was provided with coded samples to avoid bias. The scoring was done according to the industry standard (YC/T 138-1998) on a 9-point scale. The scoring was done for the seven attributes, including aroma quality, aroma quantity, harmony, irritation, aftertaste, smoothness, and combustibility.

#### 2.6.2 Analysis of degradation products in OGL-treated upper flue-cured tobacco leaves (Zhongyan 100)

Upper flue-cured tobacco leaves (Zhongyan 100) treated with 1% enzyme for 6 h were dried, ground, and sieved (80-mesh) to obtain fine powder. Pectin content was measured using the carbazole-sulfuric acid method with galacturonic acid as the standard (Kianoush et al., 2023). Galacturonic acid and glucose were quantified by high-performance liquid chromatography with evaporative light scattering detection (HPLC-ELSD). Separations were performed on an XBridge BEH Amide column; column temperature: 30 °C; flow rate: 1.0 mL/min; run time: 30 min; injection volume: 5 µL. The column was equilibrated with the mobile phase for at least 30 min before sample injection. The ELSD settings included a nitrogen nebulizer gas flow of 2.2 L/min and an injection volume of 5 μL. Oligogalacturonide (OG) content was determined spectrophotometrically.

#### 2.6.3 Chemical component analysis of tobacco powder treated with OGL

To elucidate the chemical mechanism underlying the enhanced sensory quality resulting from OGL treatment, chemical component analysis of the tobacco powder was performed using GC-MS. The enzyme-treated tobacco powder (40 mesh) was homogenized with 10 mL of PBS buffer through vortex mixing. After a 5-min incubation, 10 mL of acetonitrile and 50 μL of 2,6-dichlorotoluene (used as an internal standard) were added, followed by vortex mixing and freezing at −20 °C for 30 min. The mixture was then extracted with 1.5 g of NaCl, 6 g of anhydrous MgSO_4_, and 5 mL of dichloromethane. After thorough mixing, the sample was centrifuged at 8,000 × *g* for 3 min. The organic phase was dried over anhydrous MgSO_4_, vortexed, and then centrifuged again. Finally, 1 mL of the supernatant was filtered and analyzed using GC-MS analysis under the conditions described by Huang et al. (2022).

GC-MS analysis was performed under the following conditions. GC: HP-5MS column (60 m × 0.25 mm × 0.25 μm); helium carrier gas; injection volume: 1 μL; injector temperature: 230 °C; flow rate: 1.0 mL/min; split ratio: 10:1; solvent delay: 10 min. The oven temperature was programmed as follows: 50 °C, increased at 4 °C/min to 280 °C, and held for 10 min. MS: Transfer line temperature: 270 °C; ion source temperature: 230 °C; quadrupole temperature: 150 °C; ionization mode: EI; electron energy: 70 eV; mass scan range: *m/z* 35–550.

#### 2.6.4 Chemical compounds analysis of tobacco extract treated with OGL

To explore the effect of OGL on tobacco extracts and evaluate the potential of OGL for the biovalorization of low-value tobacco resources, chemical component analysis was performed using GC-MS on tobacco extract from lower-position Zhongyan 100 leaves with midribs. A 500 μL aliquot of OGL was incubated with 2 mL of sterile tobacco extract (50% w/v, autoclaved at 120 °C) at 45 °C with shaking at 180 × rpm for 6 h—a heat-inactivated enzyme served as the control. After incubation, 2 mL of ethyl acetate and 50 μL of 2,6-dichlorotoluene (internal standard) were added, followed by vortexing at 2,500 × rpm for 5 min and centrifugation at 10,000 × *g* for 3 min. The organic phase was dried over anhydrous sodium sulfate for more than 6 h, filtered through a 0.22 μm membrane, and analyzed by GC-MS as previously described (Huang et al., 2022).

#### 2.6.5 Statistical analysis

Data were analyzed using Microsoft Excel 2016 and SPSS 27.0. Statistical significance was assessed using one-way ANOVA followed by Tukey’s HSD *post hoc* test for multi-group comparisons. For pairwise comparisons (treated vs. control), independent samples t-tests were applied. A p-value <0.05 was considered statistically significant. All analyses were performed using SPSS 27.0 and Graphs were generated with Origin 2024. In addition to the sensory evaluation (n = 7), all experiments were conducted in triplicate (n = 3), with results expressed as mean ± standard deviation (SD).

## 3 Results

### 3.1 Construction of the recombinant OGL expression vector

**FIGURE 1:**
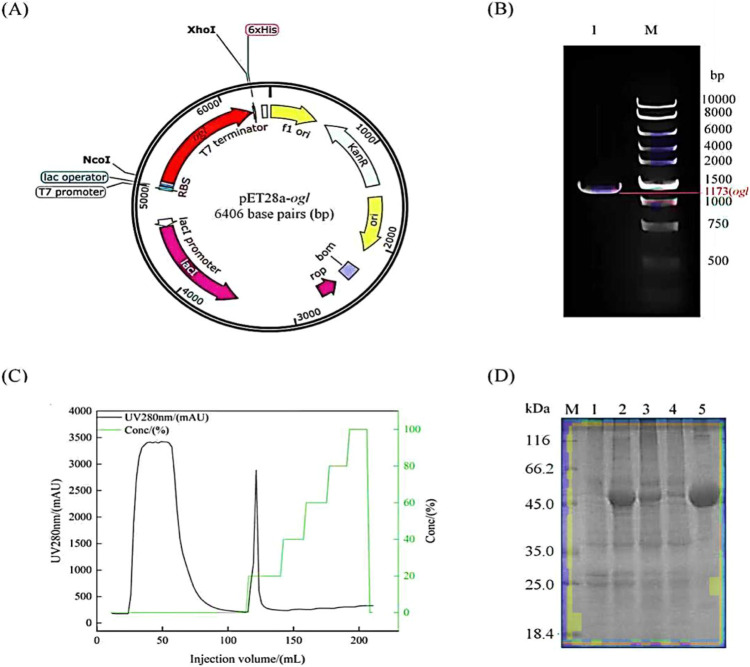
Construction and verification of recombinant pET-28a-*ogl* plasmid, and expression and purification of recombinant OGL. **(A)** Schematic map of the recombinant plasmid pET28a-OGL; **(B)** Colony PCR analysis of *E. coli* BL21 (DE3) transformants. Lane 1: PCR product showing the expected amplification band for OGL; Lane M: DNA size marker (DNA ladder); The theoretical molecular weight of the target band is 1173 bp. **(C)** Chromatographic profile of recombinant OGL purified using nickel-affinity chromatography. The UV absorbance at 280 nm (left Y-axis) and the Buffer B concentration gradient (right Y-axis) are plotted against the elution volume. The initial flow-through peak represents unbound proteins, while the sharp peak eluted at 20% Buffer B indicates the target His-tagged OGL protein. **(D)** SDS-PAGE analysis of recombinant OGL expression and purification. Lane M contains the protein molecular weight marker. Lane 1 (uninduced lysate) shows no visible band at the expected size. In contrast, Lane 2 (induced lysate) displays a distinct band at ∼47 kDa. Lane 3 (crude enzyme extract) also shows the target protein band, verifying its solubility. Lane 4 represents the flow-through fraction (0% Buffer B, in which minimal protein is visible at the target size, suggesting effective binding to the nickel affinity column. Finally, Lane 5 shows the purified protein eluted with 20% Buffer B, revealing a strong, sharp band with minimal contaminants, which confirms the successful purification of recombinant OGL with high purity.

The *ogl* gene-containing plasmid was transformed into *E. coli* BL21 (DE3) (Figure 1A). The positive transformants grown on kanamycin-containing LB plates were confirmed through colony PCR. The electrophoretogram of colony PCR showed clear bands corresponding to the *ogl* gene size (1,173 bp) (Figure 1B). The sequencing of the *ogl-*containing plasmid confirmed the sequence integrity of *ogl*. The sequencing data, including the chromatograms and the aligned sequence (Supplementary Figure S1), have been deposited in the Supplementary Material S4.

### 3.2 Expression and purification of recombinant OGL

The expression of recombinant OGL in *E. coli* BL21 (DE3) was verified by SDS-PAGE analysis. No clear band corresponding to the size of the OGL protein was observed in the cell lysate before induction; however, after IPTG induction, a distinct band corresponding to OGL (∼47 kDa) was clearly detected (Figure 1D). The expression conditions for recombinant OGL were optimized, and the highest yield of soluble, properly folded OGL was obtained under the following conditions: (i) induction at an OD_600_ of 0.8–1.0; (ii) an IPTG concentration of 1 mM; (iii) and incubation at 16 °C for 16 h. These mild induction conditions align with previous findings that lower temperatures enhance proper protein folding and solubility (Sassi et al., 2017; Mi-Ran and Pil, 2020; Arshpreet et al., 2021).

The results of nickel-affinity chromatography showed two distinct absorbance peaks at 280 nm (Figure 1C). The first peak is a flow-through peak collected at 0% Buffer B, and the second was an OGL protein peak eluted with 20% Buffer B, reaching a maximum absorbance of 2,813 mAU (Figure 1C). The SDS-PAGE analysis confirmed the production and purity of recombinant OGL protein, as a single band corresponding to OGL (approximately 47 kDa) appeared on the gel (Figure 1D). The protein concentration was 18.63 mg/mL, and the purity was approximately 92%, as determined by densitometric analysis.

The overall yield was approximately 246 mg of purified protein per liter of bacterial culture, demonstrating that the *E. coli* BL21 (DE3) expression system is highly efficient and suitable for producing functionally active OGL protein derived from *K. variicola* GB3.

### 3.3 Biochemical characterization of recombinant OGL

#### 3.3.1 Substrate specificity of recombinant OGL

The substrate specificity of OGL was assessed spectrophotometrically using substrates from four distinct categories: natural pectins (tobacco pectin, pectin), α-1,4-linked galacturonides (GalA_3_, GalA_4_, PGA), and nitrophenyl glycoside analogs (PNPG^1^–PNPG^4^). Enzymatic activity toward the pectic and galacturonide substrates was measured at 235 nm, while activity toward the PNPG analogs was determined by monitoring PNP release at 405 nm. Additionally, to illustrate structural differences and support activity analysis, a schematic diagram of the tested substrates is provided in Supplementary Figure S2.

**FIGURE 2:**
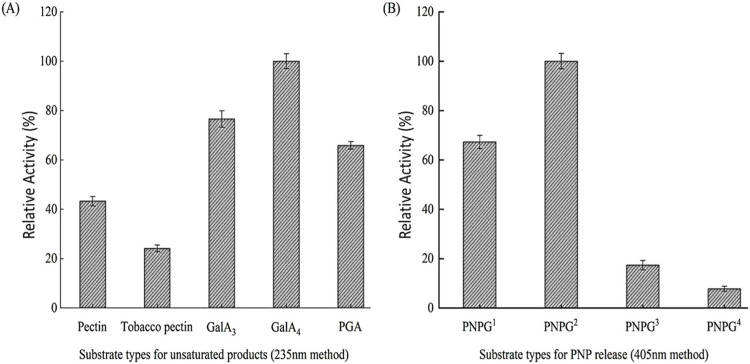
Substrate specificity of recombinant OGL. **(A)** OGL activity on substrates producing unsaturated bonds (assayed at 235 nm). Relative activities were calculated with GalA_4_ as the reference (original activity = 176.26 μmol/min/mg, set as 100% activity). **(B)** OGL activity on PNP-releasing substrates (assayed at 405 nm). Relative activities were calculated with PNPG^2^ as the reference (original activity = 8.31 μmol/min/mg, set as 100% activity). Data are presented as mean ± SD of three independent replicates (n = 3). Note that original activities obtained by the two methods are not directly comparable due to distinct detection principles; The relative activities are intended solely for comparing substrates within the same assay system.

The substrate specificity of recombinant OGL is closely linked to its catalytic mechanism and the structural features of the substrates. OGL demonstrated a clear substrate preference for oligogalacturonides (see Figure 2A). Assayed at 235 nm, it catalyzed the cleavage of GalA_4_ via a β-elimination mechanism most efficiently (set as 100% activity), with lower activity toward GalA_3_, PGA, pectin, and tobacco pectin. Significantly lower activity was observed for tobacco pectin and pectin compared with oligomeric substrates, with tobacco pectin showing lower activity than commercial pectin. The preference for GalA4 over GalA3 is consistent with findings for homologous OGL enzymes, in which extended substrate chains engage additional subsites in the active site cleft, thereby improving binding affinity (Shevchik et al., 1999; Abbott et al., 2010). Structural or docking analyses would be required to confirm this mechanism for the *K. variicola* OGL, and such studies are planned as future work. Activity toward PGA, tobacco pectin, and pectin confirms its efficient cleavage of α-1,4-glycosidic bonds via a β-elimination mechanism. However, activity toward tobacco pectin was relatively low compared to commercial pectin, which is attributed to its higher degree of esterification (Ayala-López et al., 2020; Cao et al., 2025) and structural complexity resulting from extraction methods. Despite this, OGL retained 27.18% activity toward highly esterified tobacco pectin relative to GalA_4_, demonstrating its capacity to degrade structurally complex native substrates.

OGL showed negligible activity toward the artificial PNP-releasing substrates. These substrates differ structurally from natural galacturonides in their core sugar units (e.g., galactose or glucuronic acid), as illustrated in Supplementary Figure S2. As shown in Figure 2B, activity assays monitored at 405 nm revealed that OGL exhibited the highest activity toward PNPG^2^ among the tested nitrophenyl glycosides. However, progressively lower activities were detected for PNPG^1^, PNPG^3^, and PNPG^4^. In contrast, OGL showed markedly low activity (relative activity < 20%) against the nitrophenyl glycosides PNPG^3^ and PNPG^4^, likely due to the structural dissimilarity of their core sugar units compared to galacturonic acid.

In conclusion, the substrate specificity analysis demonstrates that OGL exhibits a strong catalytic preference for oligogalacturonide substrates, particularly GalA_4_, over synthetic nitrophenyl-glycoside substrates. Based on its highest original activity among all tested substrates, GalA_4_ was selected for all subsequent characterization of OGL’s enzymatic properties.

#### 3.3.2 Effects of temperature and pH on the activity of recombinant OGL

**FIGURE 3:**
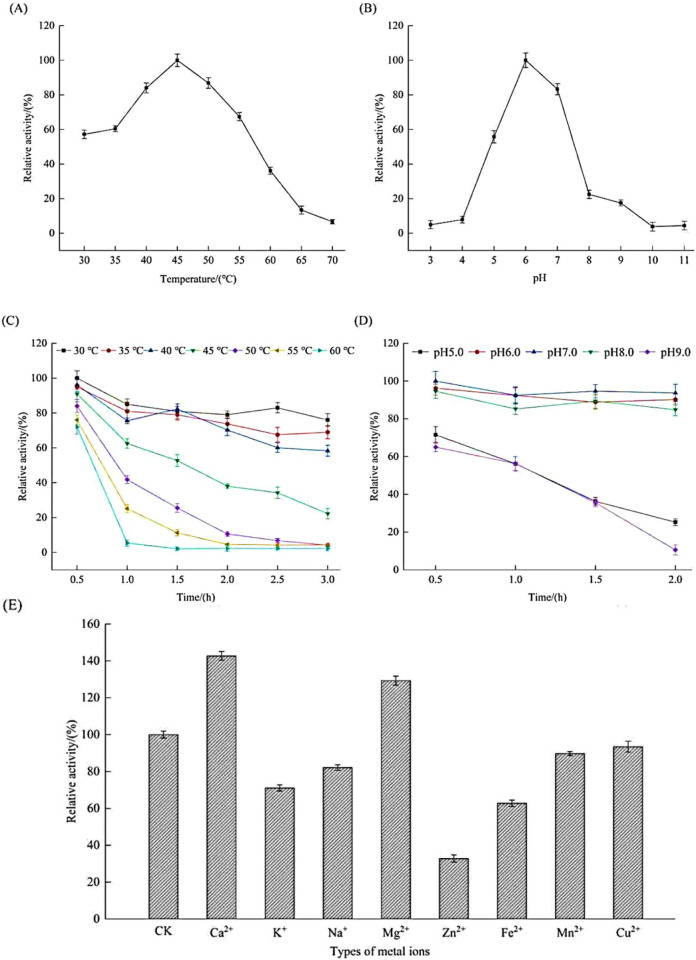
Characterization of the enzymatic properties of recombinant OGL. **(A)** Temperature profile of OGL activity. The enzyme exhibited optimal activity at 45 °C, with substantial activity retained between 40 °C and 50 °C. Activity sharply declined above 55 °C, indicating thermal sensitivity at higher temperatures. **(B)** pH profile of OGL activity. Maximum enzymatic activity was observed at pH 6.0, with significant activity maintained between pH 5.5 and 7.5. Activity decreased markedly under more acidic or alkaline conditions. **(C)** Temperature stability analysis of recombinant OGL under different temperatures. The thermal stability of OGL was examined during a 3-h incubation at temperatures ranging from 30 °C to 60 °C. The enzyme remained highly stable at 30 °C and 40 °C, but its activity dropped sharply at temperatures above 50 °C, showing heat sensitivity. **(D)** pH stability analysis of recombinant OGL under different pHs. The pH stability profile of OGL was tested over a 2-h incubation period at pH levels ranging from 5.0 to 9.0. The enzyme maintained high activity between pH 6.0 and 7.0, with notable loss of activity at more acidic (pH 5.0) and alkaline (pH 9.0) levels. **(E)** Effect of different metal ions (10 mmol/L) on the enzymatic activity of recombinant OGL. Ca^2+^, Mg^2+^, and K^+^ significantly increase enzyme activity, with Ca^2+^ showing the strongest activation. In contrast, Zn^2+^ and Fe^2+^ display strong inhibitory effects, while Na^+^ and Cu^2+^ cause moderate inhibition. The control (CK) was measured without the addition of metal ions. Error bars indicate standard deviations from triplicates. Data are presented as the mean ± SD (n = 3).

To determine the optimal temperature, the enzyme was incubated with 1% (w/v) GalA_4_ in a pH 7.5 buffer at different temperatures for 30 min. For pH, the assay was performed under the optimal temperature using buffers of different pH values, followed by activity measurement. The effects of temperature and pH on the activity of OGL are shown in Figures 3A,B. Under different temperature conditions, recombinant OGL demonstrated strong enzymatic activity. When the temperature increased from 30 °C to 45 °C, the enzyme activity gradually increased; however, when the temperature further rose from 45 °C to 70 °C, the activity sharply declined. Therefore, the optimal temperature for recombinant OGL activity was identified as 45 °C. Between 40 °C and 50 °C, the enzyme activity stayed at or above 80% of its maximum, but at temperatures between 60 °C and 70 °C, the relative activity dropped below 50%, indicating that the enzyme is not thermotolerant.

The relationship between recombinant OGL activity and pH is shown in Figure 3B. When the pH was between 3.0 and 4.0, enzyme activity was very low; when the pH exceeded 4.0, enzyme activity increased sharply. At a pH of 6.0, enzyme activity reached its maximum; however, as the pH continued to rise beyond this point, enzyme activity began to decline. When the pH was between 10.0 and 11.0, enzyme activity was again very low. Therefore, the optimal pH for recombinant OGL is 6.0, indicating that it is a weakly acidic enzyme. The optimal reaction conditions for recombinant OGL are similar to those of polygalacturonan lyase.

#### 3.3.3 Stability study of recombinant OGL

To evaluate OGL’s pH and temperature stability, the enzyme was incubated at various temperatures (at optimal pH) or pH conditions (at optimal temperature) for different durations. Residual activity was measured after adding 1% GalA_4_. The thermal stability of recombinant OGL was evaluated, and the results are shown in Figure 3C. The OGL enzyme remained highly stable at 30 °C and 40 °C during a 3-h incubation; on the other hand, its activity rapidly decreased at 50 °C and 60 °C, perhaps because the higher temperatures disrupted the structure of OGL. The thermal stability analysis revealed that OGL has a half-life of 1.51 h at 45 °C. It should be noted that the t_1/2_ was measured under dilute buffer conditions; in the tobacco matrix, structural effects from the substrate environment may modulate enzyme stability. The enzyme remained stable over a pH range of 6.0–8.0 for 2 h (Figure 3D). In contrast, enzyme activity sharply decreased at pH 5.0 and pH 9.0. This indicates that both acidic and alkaline conditions negatively impact enzyme stability. The enzyme’s thermal stability and broad pH tolerance collectively ensure reliable performance under typical industrial conditions.

#### 3.3.4 Effect of metal ions on the activity of recombinant OGL

The results of incubating OGL in buffers containing various metal ions (final concentration 10 mmol/L) with 1% GalA_4_ for 30 min showed that K^+^, Ca^2+^, and Mg^2+^ significantly increased enzyme activity, whereas Zn^2+^, Fe^2+^, and Cu^2+^ had inhibitory effects (Figure 3E). Notably, Ca^2+^ showed the strongest activation effect, suggesting a possible role in enzyme activation consistent with known Ca^2+^-mediated mechanisms in structurally related pectate lyases, where Ca^2+^ has been shown to facilitate catalysis by modulating active-site residue pKa values and stabilizing substrate binding (Li and Tian, 2024; Liu et al., 2024). However, confirmation of this mechanism would require structural or binding studies, which are planned as future work.

#### 3.3.5 Enzymatic kinetics of recombinant OGL

The enzymatic kinetics of OGL using GalA_4_ as the substrate were determined using by non-linear regression. The *Km* and *Vmax* of the OGL enzyme were calculated as 3.89 ± 0.18 mM and 36.41 ± 1.20 μmol/min/mg, respectively, with an *R* ^2^ value of 0.9935, indicating excellent goodness-of-fit. These results demonstrate that OGL has high substrate affinity and catalytic efficiency toward GalA_4_. The Km and Vmax of the OGL enzyme were determined as 4.34 mM and 38.8 μmol/min/mg, respectively, indicating moderate substrate affinity comparable to previously reported OGL and pectate lyase enzymes (Km range: 1.5–9 mM) (Abbott et al., 2010; Zhao et al., 2018; Liu et al., 2024).

### 3.4 Functional and application studies of recombinant OGL

#### 3.4.1 Analysis of degradation products in OGL-treated upper flue-cured tobacco leaves (Zhongyan 100)

Treatment of tobacco leaves with 1% OGL significantly reduced the pectin content by 32.18% (Table 1). The contents of galacturonic acid and oligogalacturonides increased significantly by 39.86% and 403.85%, respectively, while glucose levels remained unchanged compared with the control. These results confirm the specific and effective degradation of pectin by OGL. Although OGL has a half-life of 1.51 h at 45 °C under buffer conditions, the enzyme retains substantial cumulative activity over the 6-h incubation period. This is supported by the significant pectin degradation (32.18%) observed during incubation. Enhanced aroma compound formation further confirms that the enzyme remained functionally active throughout the process.

**TABLE 1:** Enzymatic hydrolysis products and conversion rates in upper flue-cured tobacco leaves (Zhongyan 100).

Compound	Control/(mg/g)	Enzyme-treated/(mg/g)	Change rate/(%)
Pectin	16.72 ± 0.84^b^	11.34 ± 0.57^a^	32.18↓
Galacturonic acid	12.82 ± 0.42^b^	17.93 ± 0.86^a^	39.86↑
Glucose	2.08 ± 0.10^a^	2.16 ± 0.13^a^	3.85↑
OGs	0.26 ± 0.04^b^	1.31 ± 0.15^a^	403.85↑

All experiments were conducted using enzyme-free tobacco leaves as blank controls, with three biological replicates per group. Results are presented as mean ± standard deviation (SD). Lowercase letters (a, b) denote statistical significance; identical letters indicate no significant difference, while different letters indicate significant differences at p < 0.05.

#### 3.4.2 Sensory evaluation of tobacco strands from Zhongyan 100 treated with OGL

**FIGURE 4:**
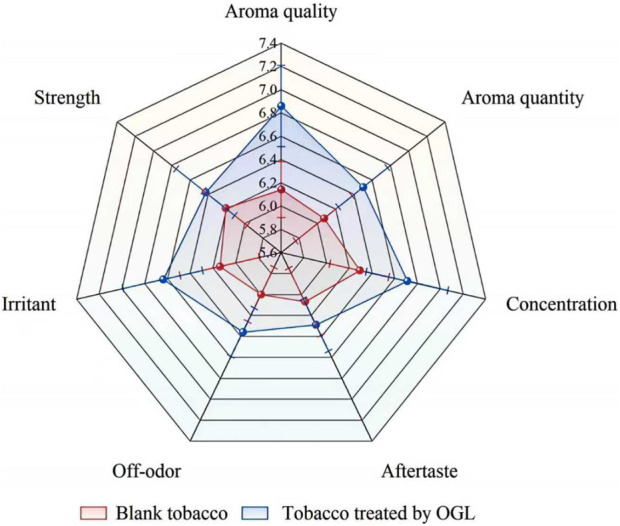
Sensory evaluation of Zhongyan 100 tobacco strands treated with recombinant OGL. Scores are shown as mean ± SD (n = 7). For negative sensory attributes (pungency, irritation, off-flavor), scores are assigned inversely on the 9-point scale (YC/T 138-1998), such that higher scores indicate reduced undesirable attributes (i.e., improvement).

To assess the functional impact of recombinant OGL on tobacco product quality, a sensory evaluation using a double-blind design was conducted on Zhongyan 100 tobacco strands treated with the enzyme. As shown in Figure 4, the enzyme-treated strands displayed increased aroma quality and aroma quantity, and a noticeable reduction in off-odor, intensity, and irritation during smoking. For negative sensory attributes (pungency, irritation, off-flavor), scores are assigned inversely on the 9-point scale (YC/T 138-1998), such that higher scores indicate reduced undesirable attributes (i.e., improvement). Detailed sensory evaluation data are presented in Supplementary Table S1. The improvement in aroma quality results from the enzymatic degradation of pectin-like polysaccharides. Based on established biochemical pathways, the degradation products—including reducing sugars and galacturonic acid liberated from pectin hydrolysis—may participate in Maillard-type reactions during tobacco curing and processing, potentially contributing to aroma formation, including the generation of furans and caramel-like volatile compounds (Weng et al., 2024). However, direct experimental verification of this pathway was not conducted in the present study and warrants future investigation. These reactions can produce aroma-active compounds and flavor precursors, thereby increasing the sensory complexity of the final product. Sensory results aligned with enzymatic hydrolysis products from Zhongyan 100 leaves. The degradation products—OGs (which may contribute to the reduction of green-note compounds) and galacturonic acid (aroma precursor)—jointly enhanced sensory quality. Overall, the marked enhancement in sensory attributes suggests that recombinant OGL has strong potential for application in tobacco processing, enhancing product quality and consumer satisfaction.

#### 3.4.3 Analysis of chemical components of tobacco powder treated by OGL

To explore the chemical mechanism underlying the enhancement of sensory quality in OGL-treated cigarettes, chemical component analysis was performed on tobacco powder before and after treatment. The effects of enzymatic treatment on the aroma components of tobacco powder are shown in Table 2. After treatment, the levels of 19 aroma compounds markedly increased, with 9 compounds increasing by 15%–30% and another eight compounds increasing by more than 30%. Notably, four compounds—solanone, 4,7,9-Megastigmatrien-3-one, dihydroactinidiolide, and loriolide—showed particularly significant contributions to the aromatic profile of smoke, with increases of 43.95%, 62.86%, 47.37%, and 37.44%, respectively. However, six aroma compounds exhibited a significant reduction after treatment, with the decrease ranging from 15% to 50%.

**TABLE 2:** Comparison of chemical components of tobacco powder before and after OGL treatment.

Num	Compound	Control/(µg/mL)	Enzyme-treated/(µg/mL)	Change rate/(%)
1	Furfural	2.76 ± 0.17^b^	3.78 ± 0.42^a^	36.96↑
2	5-Hydroxymethylfurfural	1.84 ± 0.11^b^	2.21 ± 0.18^a^	20.11↑
3	Phenylacetaldehyde	5.92 ± 0.31^b^	7.78 ± 0.70^a^	31.42↑
4	Solanone	4.05 ± 0.38^b^	5.83 ± 0.44^a^	43.95↑
5	4,7,9-Megastigmatrien-3-one	3.85 ± 0.22^b^	6.27 ± 0.36^a^	62.86↑
6	Phytone	0.97 ± 0.05^b^	1.31 ± 0.16^a^	35.05↑
7	Geranylacetone	1.75 ± 0.13^b^	2.14 ± 0.19^a^	22.29↑
8	4-Hydroxy-β-dihydrodamascenone	5.40 ± 0.31^a^	6.18 ± 0.48^a^	14.44↑
9	3-Hydroxy-7,8-dihydro-β-ionol	4.49 ± 0.25^b^	5.71 ± 0.53^a^	27.17↑
10	2-Methyldecanol	0.92 ± 0.06^b^	1.12 ± 0.14^a^	21.74↑
11	5,6-Dihydro-2H-pyran-2-one	1.48 ± 0.18^b^	1.95 ± 0.24^a^	31.76↑
12	4-Hydroxyphenethyl alcohol	1.63 ± 0.11^a^	1.87 ± 0.22 ^a^	14.72↑
13	2-Butyl-1-octanol	2.74 ± 0.21^b^	3.44 ± 0.34^a^	25.55↑
14	2-Propyl-1-heptanol	7.43 ± 0.55^b^	8.55 ± 0.77^a^	15.07↑
15	Dihydroactinidiolide	1.52 ± 0.10^b^	2.24 ± 0.27^a^	47.37↑
16	Loriolide	2.11 ± 0.15b	2.90 ± 0.32^a^	37.44↑
17	Phytyl acetate	0.93 ± 0.07^a^	1.07 ± 0.13^a^	15.05↑
18	Scopoletin	0.87 ± 0.06^b^	1.10 ± 0.12^a^	26.44↑
19	Oxyeugenol acetate	3.26 ± 0.26^b^	3.98 ± 0.48^a^	22.09↑
20	Citric acid	5.72 ± 0.31^a^	3.91 ± 0.27^b^	31.64↓
21	Acetic acid	3.26 ± 0.19^a^	2.17 ± 0.12^b^	33.44↓
22	Nicotine	1.69 ± 0.11^a^	1.35 ± 0.09^b^	20.12↓
23	n-Butane	2.04 ± 0.15^a^	1.69 ± 0.18^b^	17.16↓
24	Pentadecane	1.61 ± 0.20^a^	1.36 ± 0.12^b^	15.53↓
25	1-Pentadecene	1.11 ± 0.13^a^	0.58 ± 0.05^b^	47.75↓

Data are presented as the mean ± SD, of three independent experiments. The change rate is calculated from two sets of mean values. Lowercase letters (a, b) denote statistical significance; identical letters indicate no significant difference, while different letters indicate significant differences at p < 0.05.

The increase of furan derivatives such as furfural and 5-hydroxy methyl furfural is due to the release of galacturonic acid (furfural precursor), released by OGL by degrading tobacco pectin, which contributes to the caramelization characteristics of flue gas. Appropriate furfural can mitigate green notes in tobacco leaves and, through synergy with flavor compounds like organic acids and esters, yield smoother smoke with reduced harshness. Loriolide, a natural lactone, provides fruity and faint floral notes. Solanone strengthens the fermented flavor, and 4,7,9-Megastigmatrien-3-one improves mildness. The increase in phytone (35.05%), a potent ketone fragrance derived from carotenoid degradation, suggests a broader enzymatic impact on the terpenoids. Phytone contributes significantly to the base notes and overall aromatic complexity of tobacco, with its mild, sweet, and hay-like scent. Phenylacetaldehyde and 4-hydroxy-β-dihydrodamascenone jointly provide violet and rose-like floral notes. These compounds synergistically shaped the complex and unique aromatic profile of tobacco (Muradova et al., 2023).

Additionally, OGL treatment of upper Zhongyan 100 tobacco leaves led to notable reductions in several compounds. Citric acid dropped by 31.64%, because OGs may influence intracellular pH and potentially promote the transformation of organic acids into flavor compounds such as esters. Acetic acid decreased by 33.44%, which lessens the pungency and irritation of the smoke. Nicotine decreased slightly (20.12%), which may contribute to reduced toxicity and harshness. The decreases were observed in alkanes and alkenes, including n-butane, pentadecane, and 1-pentadecene. Cyclododecane and guaiene were undetectable in the treated group. These changes suggest that OGL improves the overall aroma profile of tobacco by reducing undesirable off-flavors and irritation, thus enhancing smoking quality.

In conclusion, OGL effectively improves the overall quality of tobacco under the tested conditions by structurally modifying the plant matrix and releasing key flavor compounds. These findings provide a robust rationale for employing OGL as a biocatalyst to valorize tobacco resources.

#### 3.4.4 Analysis of chemical components of tobacco extracts treated by OGL

To explore the effect of OGL on tobacco extracts, GC–MS analysis was performed on tobacco extracts before and after fermentation. As shown in Table 3, the levels of several key aroma compounds-furfural, solanone, β-damascone, 4,7,9-germacratrien-3-one, dihydro-β-ionol, and loriolide-increased significantly following enzymatic treatment, with relative increases ranging from 31.45% (furfural) to 80.87% (4,7,9-germacratrien-3-one). These compounds are known to contribute to sweet, floral, and woody aromas. OGL may contribute to increased β-damascenone formation by facilitating cell wall disruption and release of carotenoid precursors, which are subsequently converted into β-damascenone. Additionally, OGL treatment enhances neophytadiene in tobacco extract by degrading cell walls, facilitating the release of sequestered neophytadiene.

**TABLE 3:** Comparison of the chemical components in tobacco extracts before and after OGL treatment.

Num	Compound	Control/(µg/mL)	Enzyme- treated/(µg/mL)	Change rate/(%)
1	Furfural	2.71 ± 0.24^b^	4.62 ± 0.41^a^	70.48↑
2	Furfuryl alcohol	2.88 ± 0.26^b^	3.35 ± 0.30^a^	16.32↑
3	4,7,9-Germacratrien-3-one	11.50 ± 1.04^b^	20.80 ± 1.87^a^	80.87↑
4	Ethyl maltol	5.15 ± 0.46^b^	6.50 ± 0.58^a^	26.21↑
5	β-Damascenone	0.61 ± 0.05^b^	0.85 ± 0.08^a^	39.34↑
6	Dihydro-β-ionol	0.71 ± 0.06^b^	1.20 ± 0.11^a^	69.01↑
7	Dihydroactinidiolide	2.98 ± 0.27^b^	4.40 ± 0.39^a^	47.65↑
8	β-Damascone	0.40 ± 0.03^b^	0.69 ± 0.06^a^	72.50 ↑
9	Vanillin	3.65 ± 0.33^b^	5.40 ± 0.48^a^	47.95↑
10	Germacrone	11.70 ± 1.05^b^	15.80 ± 1.42^a^	35.04↑
11	4-Hydroxy-β-dihydrodamascenone	2.33 ± 0.21^b^	3.32 ± 0.30^a^	43.78↑
12	Loriolide	1.02 ± 0.09^a^	1.65 ± 0.15^a^	61.76↑
13	Undecanol	3.10 ± 0.28^b^	4.50 ± 0.40^a^	45.16↑
14	Solanone	15.50 ± 1.40^b^	26.50 ± 2.38^a^	70.97↑
15	2-Methyldecanol	1.65 ± 0.15^b^	2.34 ± 0.21^a^	41.82↑
16	Neophytadiene	2.33 ± 0.21^b^	3.42 ± 0.27^a^	46.78↑
17	Phytol	7.08 ± 0.64^a^	8.30 ± 0.75^a^	17.23↑
18	cis-3-Hexen-1-ol	4.20 ± 0.38^a^	2.65 ± 0.24^b^	36.90↓
19	p-Isopropylphenol	2.70 ± 0.24^a^	1.50 ± 0.13^b^	44.44↓
20	Acetic acid	5.88 ± 0.53^a^	3.40 ± 0.30^b^	42.18↓
21	Palmitic acid	2.68 ± 0.24^a^	2.05 ± 0.18^b^	23.51↓
22	n-Butane	1.01 ± 0.09^a^	0.72 ± 0.06 ^b^	28.71↓
23	Linoleic acid	6.63 ± 0.42^a^	5.75 ± 0.49 ^a^	13.27↓

Data are presented as the mean ± SD, of three independent experiments. The change rate is calculated from two sets of mean values. Lowercase letters (a, b) denote statistical significance; identical letters indicate no significant difference, while different letters indicate significant differences at p < 0.05.

Following OGL treatment, levels of undesirable compounds in the tobacco extract were significantly reduced. Green-note volatiles cis-3-Hexen-1-ol decreased due to inhibition of biosynthesis by OGs derived from pectin degradation. Phenolic compounds slightly declined, with a significant reduction (44.44%) in the astringent p-isopropylphenol. Reduction of acetic acid lowered irritancy and enhanced the overall aroma. These changes demonstrate OGL’s efficacy in improving extract quality.

The increase in flavor components was mainly mediated by pectin degradation via oligogalacturonide lyase activity. This process disrupts the tobacco cell wall structure to release free flavor substances and indirectly facilitates the release and conversion of bound flavor precursor compounds. Furthermore, OGL may indirectly facilitate the release of glycoside-bound aroma precursors and enhance substrate availability for characteristic tobacco aroma formation. Analysis of tobacco extract from lower-value leaves demonstrated that OGL treatment effectively modified its chemical composition, supporting its potential to upgrade lower-quality tobacco materials and create value from agricultural waste streams.

## 4 Discussion

To the best of our knowledge, this is the first report of heterologous expression of OGL derived from *K. variicola* GB3 in *E. coli*, along with its functional characterization and application analysis. OGL exhibited a higher substrate preference for GalA_4_ and GalA_3_, consistent with the activities of previously reported OGL enzymes (Chatterjee et al., 1985). However, it preferentially cleaves longer-chain substrates such as GalA_4_ compared with GalA_3_. It also effectively cleaved α-1,4-glycosidic bonds in PGA and pectins. The relatively lower activity toward native tobacco pectin can be attributed to its higher degree of esterification and structural complexity. In contrast, negligible activity was observed against artificial PNP substrates, likely due to the absence of a galacturonate backbone compatible with the β-elimination catalytic mechanism of OGL. The biochemical characterization demonstrated that OGL exhibits distinct functional traits compared with other members of its enzyme family, including a mildly acidic pH optimum (6.0) and a moderate temperature optimum (45 °C). These features make it suitable for industrial applications (Cheng et al., 2017; Zhou et al., 2017; Liu et al., 2022; Liu et al., 2024; Zhou et al., 2024). OGL also exhibited broad pH stability and moderate thermal stability (30 °C–40 °C), with a half-life (t_1_/_2_) of 1.51 h at 45 °C, reflecting a stability profile typical of mesophilic microbial enzymes (Yehui et al., 2023). OGL is activated by Ca^2+^, a characteristic that distinguishes it from several homologous enzymes that exhibit metal-induced inhibition. A similar characteristic has been reported for pectin lyases derived from *Bacilus velezensis*, whose activity was significantly enhanced by 5 mmol/L Ca^2+^ (Li and Tian, 2024; Li et al., 2024). The kinetic parameters (*K*_*m*_ = 3.89 ± 0.18 mM and *V*_max_ = 36.41 ± 1.20 μmol/min/mg) indicate that OGL demonstrates effective substrate binding affinity and catalytic efficiency toward GalA_4_ relative to other tested substrates. Direct comparison with previously reported pectin lyases is not appropriate due to differences in substrate types and assay methodologies (Zhao et al., 2018). But the kinetic parameters determined in this study clearly reveal the preferential recognition and cleavage of longer-chain oligogalacturonides via β-elimination by OGL.

The application-oriented experiments, such as the treatment of flue-cured tobacco leaves with OGL, demonstrated that degradation of pectin was substantially elevated up to 32.18%, which is also accompanied by marked increases in galacturonic acid (39.86%) and oligogalacturonides (403.85%) production. GC–MS profiling also revealed significant increases in key aroma compounds, including solanone (43.95%–70.97%), dihydroactinidiolide (47.37%–47.65%), β-damascone derivatives (39.34%–72.50%), and furfural (36.96%–70.48%).

Mechanistically, OGL appears to contribute to aroma enhancement through multiple pathways: (i) structural disruption of cell wall pectin networks; (ii) release of oligogalacturonides and (iii) indirect promotion of carotenoid- and Maillard-derived aroma compound formation. This multi-level biochemical effect of OGL supports its targeted application in tobacco bioprocessing (Mai et al., 2024).

The versatile catalytic profile of OGL suggests potential applications beyond tobacco processing. The application of OGL in fruit juice production (Liu et al., 2022; Pavlović et al., 2024) can improve juice clarity and extraction yield through targeted degradation of pectin networks. The application of OGL in the textile industry can offer an environmentally friendly alternative for selective degradation of pectin and consequently soften the fibre and remove the impurity (Zhou et al., 2017; Liu et al., 2024). Although this study focused on tobacco processing, the biochemical properties of OGL—including its stability at moderate pH and temperature, Ca^2+^ activation, and preference for oligogalacturonide substrates—suggest potential utility in other industries where pectinase enzymes are used, such as fruit juice clarification and textile degumming. However, these applications were not evaluated in the present study and should be investigated in future work.

Collectively, these findings not only establish *K. variicola* GB3 OGL as a functionally active and application-relevant enzyme but also provide a mechanistic framework for its integration into sustainable biotechnological strategies aimed at value enhancement of tobacco and other plant-derived materials.

## 5 Conclusion

This study successfully achieved the heterologous expression, purification, and functional characterization of a novel OGL from *K*. *variicola* GB3 in *E*. *coli*. OGL exhibited a pronounced catalytic preference for oligogalacturonides, particularly GalA_4_. OGL displayed optimal activity at pH 6.0 °C and 45 °C, along with broad pH stability (6.0–8.0) and moderate thermal stability (t_1_/_2_ = 1.51 h at 45 °C). Notably, the enzyme was significantly activated by Ca^2+^. Application-oriented evaluation demonstrated that OGL effectively degraded tobacco pectin. GC–MS profiling and sensory analysis further revealed enhanced production of key aroma-related compounds, including solanone, dihydroactinidiolide, β-damascone derivatives, and furfural. These findings establish a direct link between OGL-mediated pectin degradation and flavor enhancement in tobacco matrices.

Although further optimization is required to improve catalytic robustness for large-scale industrial applications, this study provides a mechanistic basis and a foundation for practical applications.

## Importance

To the best of our knowledge, this study presents the first report of heterologous expression and functional characterization of OGL from *Klebsiella variicola* GB3. In addition, it demonstrates OGL’s potential to reduce undesirable compounds and improve sensory quality.

## Data Availability

The original contributions presented in the study are included in the article/Supplementary Material, further inquiries can be directed to the corresponding authors.
