# Supplementary material for: A novel recombinant oligogalacturonide lyase from Klebsiella variicola promotes pectin degradation and enhances aroma formation in tobacco leaves
Source: Front Bioeng Biotechnol. 2026 Jun 16;14:1824056. doi: 10.3389/fbioe.2026.1824056 (PMC13314786; doi:10.3389/fbioe.2026.1824056)
Supplement: Supplementary file 1 [file DataSheet1.pdf]

Supplementary Material

S1 Extraction of tobacco pectin used in the study.

The tobacco pectin used in this study was extracted from a blend of ten distinct varieties of flue-cured tobacco leaves using an ultrasonic-assisted method. Specifically, 100 g of tobacco powder was mixed with 1 L of distilled water (solid-to-liquid ratio of 1:10) and subjected to ultrasonic extraction for 30 min. The resulting slurry was adjusted to pH 2.0 with 85% phosphoric acid and further ultrasonicated at 40 °C for 30 min. After cooling to room temperature, the solution was filtered through multiple layers of gauze. Pectin was precipitated from the filtrate by adding an equal volume of 95% ethanol, which resulted in the formation of a flocculent precipitate. The mixture was then centrifuged at 8000 × g for 10 min at 4 °C, after which the supernatant was discarded. The resultant yellowish gel-like pectin was transferred with a small amount of distilled water into a pre-weighed Petri dish, frozen at -80 °C for 2 h, and finally lyophilized. The weight of the dried pectin was recorded, and the product was stored at -20 °C for subsequent use.

S2 Extraction of tobacco extract used in the study

The tobacco extract used in this study was prepared from lower-canopy leaves of the Zhongyan 100 variety. The extraction process is as follows:

Dried tobacco leaves were ground and sieved through a 40-mesh screen. The resulting powder was mixed with 15% ethanol aqueous solution at a solid-to-solvent ratio of 1:10 (w/v) and extracted using ultrasound at 50 °C for 50 min to solubilize target components. Following extraction, the solution was collected and concentrated using a rotary evaporator at 50°C and a vacuum of -0.1 MPa until a concentration of approximately 40% solids was achieved. The final tobacco extract was stored at 4 °C until use.

S3 Sequence Information of the Oligogalacturonide Lyase (OGL) Gene from *Klebsiella variicola* GB3

Enzyme Name: Oligogalacturonide Lyase (OGL)

Gene Name: *ogl*

Organism： *K. variicola* GB3

Coding DNA Sequence (CDS)：

ATGGCTAAAGGCATGCGGGTCAAACTGAATTATCAGGTCAGCCACGATCCGGATAC
CGGAGCGGAAGTTACCCGCTTAACCCCTCCGGAGGTCACCTGTCATCGCAACTAC
TTCTATCAGAAGTGTTTTTTTAACGATGGCAGCCATTTACTGTTCGCCGGCGAGTTT
GACGGCCACTGGAACTACTATCTGCTGAATATCGCCAGCGCCGAGGCCATCCAGCT
GACGGAAGGCGCCGGGGATAACACCTTCGGCGGCTTCCTCTCCCCGGACGATAAG
TCGCTTTATTACGTGAAAAATGACCGCACCCTGCTGGAGGTGAATCTGACCACCCT
CGTTGAGCGCGAAGTTTATCGCGTCAGCGATGACTGGGTCGGCTACGGCACCTGG
GTGGCAAACAGCGATTGCAGCAAACTGGTGGGCATTGAGATTGCGAAAAGCGACT
GGACGCCGCTCAACGACTGGCAGATTTTCCATGACTTCTTCCACAAAGGACCGCA
CTGCCGCCTGCTGCGCGTCGACCTGCACAGCGGAGAGAGCCAGGTGATCCATGAG
GAAAAAATCTGGCTGGGACACCCGATCTATCGTCCCTTCGACGACCACACCGTCG
CCTTCTGCCATGAAGGGCCGCACGATCTGGTGGACGCCCGCATGTGGCTGGTCAAT

GAAGATGGCAGCCACGTCCGCAAAGTGAAAACGCATGCCCCGGGCGAAAGCTGC
ACCCATGAATTCTGGGTACCGGACGGTTCGGCGCTGATCTACGTCTCCTATCTGAA
AGGTCAGCAGGGGCGGACGATCTATCGCTTTGATCCCGAAAGCGGCGTCAACGAG
GCCCTGATGACCATGCCGGCCTGCTCGCATCTGATGAGCAATTTCGACGGCACGCT
GCTGGTGGGCGATGGCTCAGGGACCCCGGTGGATGTCAAAGATACCGGCGGCTAC
TCCATTGATAACGATCCTTATCTGTATGTCTTTAACGTGGCGCAAAAACGCTACTTC
CGCGTCGCGCGTCATGATACCTCGTGGGCGACGGTGGCCAACAGCCGTCAGGTGA
CTCACCCGCACCCCTCGTTTACCCCGGACGACAGCGCCATTCTGTTCAGCTCCGAT
AAAGACGGTAAACCGGCCATCTATATCGCGAAATTACCCGAGCATCCGCCAATGCT
GAGCGCCTGA(1173bp)

Amino Acid Sequence：

MAKGMRVKLNYQVSHDPDTGAEVTRLTPPEVTCHRNYFYQKCFFNDGSHLLFAGEF
DGHWNYYLLNIASAEAIQLTEGAGDNTFGGFLSPDDKSLYYVKNDRTLLEVNLTTLV
EREVYRVSDDWVGYGTWVANSDCSKLVGIEIAKSDWTPLNDWQIFHDFFHKGPHCR
LLRVDLHSGESQVIHEEKIWLGHPIYRPFDDHTVAFCHEGPHDLVDARMWLVNEDGS
HVRKVKTHAPGESCTHEFWVPDGSALIYVSYLKGQQGRTIYRFDPESGVNEALMTM
PACSHLMSNFDGTLLVGDGSGTPVDVKDTGGYSIDNDPYLYVFNVAQKRYFRVARHD
TSWATVANSRQVTHPHPSFTPDDSAILFSSDKDGKPAIYIAKLPEHPPMLSA (391aa)

Verification Note： The sequence accuracy was confirmed through DNA sequencing of the recombinant plasmid.

S4 DNA Sequencing Verification of the Recombinant OGL Expression Plasmid

The OGL sequence has been deposited in the ENA under accession number OZ351899-OZ351899. As shown in Figure S1, the sequence accuracy was confirmed through DNA sequencing of the recombinant plasmid.

In Figure S1, sequence chromatogram of the *ogl* gene insert demonstrates high-quality sequencing data with clear peak resolution. Nucleotide alignment between the reference sequence 1 and recombinant strain sequence 2 shows 100% identity across the complete coding region. The sequencing results confirm the accurate construction of the recombinant plasmid containing the complete OGL coding sequence from *K. variicola* GB3, ensuring sequence integrity for functional studies.

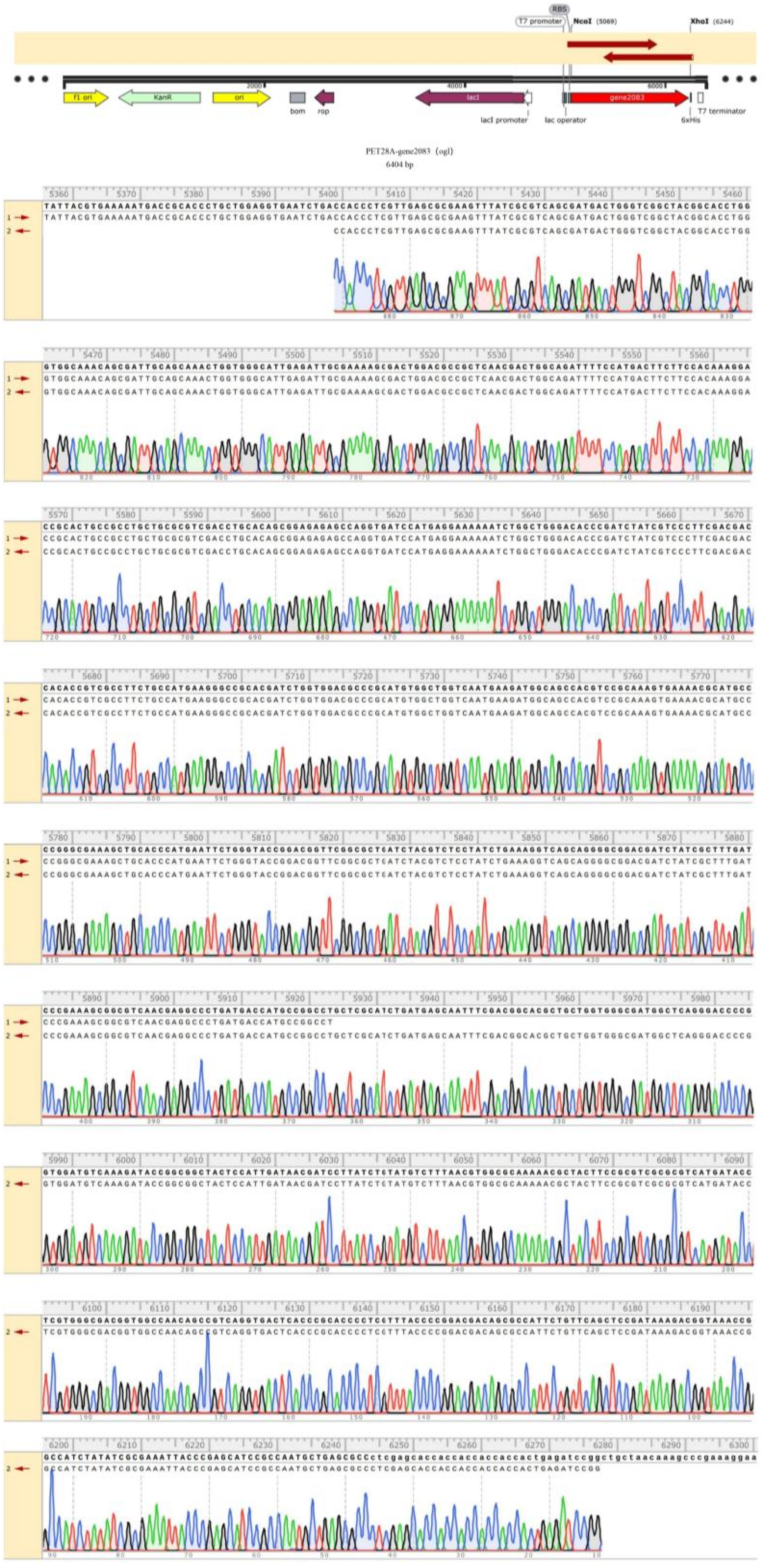

Supplementary Figure S1 DNA Sequencing Verification of the Recombinant OGL Expression Plasmid

Note: gene2083 corresponds to the *ogl* gene. Sequence analysis was performed using DNAMAN and SnapGene software.

Supplementary Figure S2

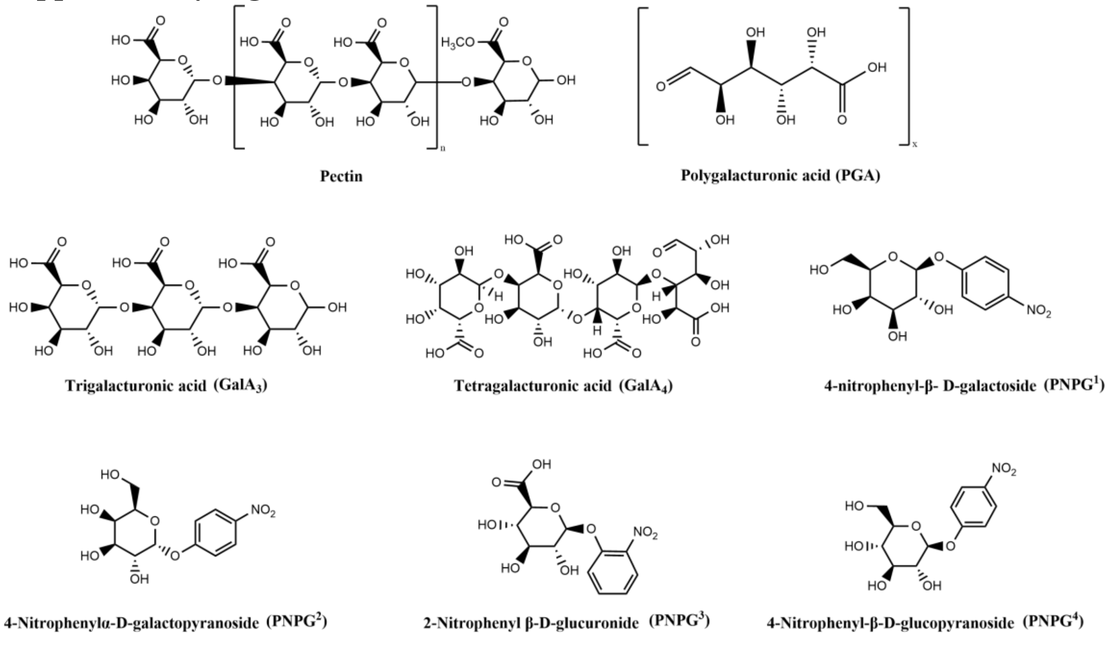

Figure S2 Chemical structures of tested substrates. Enzymatic activity was measured using nine substrates: pectin, tobacco pectin, $GalA_3$, $GalA_4$, PGA, $PNPG^1$, $PNPG^2$, $PNPG^3$ and $PNPG^4$. This figure presents a schematic diagram of the eight tested substrates to provide a structural reference and facilitate the interpretation of the observed activity differences.

Supplementary Figure Table S1 Effect of OGL fermentation on sensory quality of cigarettes (Zhongyan 100).

Parameter/(9-0)	Blank tobacco	Tobacco treated by OGL
Aroma quality	6.14±0.24^{b}	6.86±0.35^{a}
Aroma quantity	6.07±0.31^{b}	6.5±0.27^{a}
Concentration	6.29±0.26^{b}	6.71±0.36^{a}
Aftertaste	6.07±0.32^{a}	6.29±0.25^{a}
Off-odor	6.00±0.26^{b}	6.36±0.23^{a}
Irritant	6.14±0.35^{b}	6.64±0.35^{a}
Strength	6.21±0.24^{a}	6.43±0.32^{a}
Total	42.92±0.09^{b}	45.79±0.19^{a}

Note: Lowercase letters (a, b) denote statistical significance; identical letters indicate no significant difference, while different letters indicate significant differences at $p < 0.05$.
